# Spherical Lenses and Prisms Lead to Postural Instability in Both Dyslexic and Non Dyslexic Adolescents

**DOI:** 10.1371/journal.pone.0046739

**Published:** 2012-11-05

**Authors:** Zoi Kapoula, Chrystal Gaertner, Eric Matheron

**Affiliations:** IRIS Group, Centre d'Etudes SensoriMotrices UMR8194, CNRS, Université Paris Descartes, Paris, France; University of Bologna, Italy

## Abstract

There is controversy as to whether dyslexic children present systematic postural deficiency. Clinicians use a combination of ophthalmic prisms and proprioceptive soles to improve postural performances. This study examines the effects of convergent prisms and spherical lenses on posture. Fourteen dyslexics (13–17 years-old) and 11 non dyslexics (13–16 years-old) participated in the study. Quiet stance posturography was performed with the TechnoConcept device while subjects fixated a target at eye-level from a distance of 1_m. Four conditions were run: normal viewing; viewing the target with spherical lenses of −1 diopter (ACCOM1) over each eye; viewing with −3 diopters over each eye (ACCOM3); viewing with a convergent prism of 8 diopters per eye. Relative to normal viewing, the −1 lenses increased the surface of body sway significantly whereas the −3 diopter lenses only resulted in a significant increase of antero-posterior body sway. Thus, adolescents would appear to cope more effectively with stronger conflicts rather than subtle ones. The prism condition resulted in a significant increase in both the surface and the antero-posterior body sway. Importantly, all of these effects were similar for the two groups. Wavelet analysis (time frequency domain) revealed high spectral power of antero-posterior sway for the prism condition in both groups. In the ACCOM3 condition, the spectral power of antero-posterior sway decreased for non dyslexics but increased for dyslexics suggesting that dyslexics encounter more difficulty with accommodation. The cancelling time for medium range frequency (believed to be controlled by the cerebellum), was shorter in dyslexics, suggesting fewer instances of optimal control. We conclude that dyslexics achieve similar postural performances albeit less efficiently. Prisms and lenses destabilize posture for all teenagers. Thus, contrary to adults, adolescents do not seem to use efferent, proprioceptive ocular motor signals to improve their posture, at least not immediately when confronted to convergence accommodation conflict.

## Introduction

Postural control during quiet stance involves continuous multisensory central integration of visual, vestibular and proprioceptive inputs in order to produce motor commands controlling the body's position in space. Kapoula and Bucci [1] measured postural control in 13 year-old dyslexic adolescents during both closed and opened eye conditions while they were instructed to fixate a target at distances of 25 cm and 150 cm. Dyslexics were more unstable during such fixation tasks regardless of the distance at which the target was placed, be it proximal or distal. Nevertheless, when they were asked to make active vergence eye movements between the proximal and the distal target (convergence-divergence), their postural stability improved and became almost normal, while no significant change was observed in the non dyslexic control group. Moreover, a separate eye movement study [1] performed with the aid of video-oculography demonstrated marked fixation instability for dyslexics in the simple task requiring prolonged fixation. This was in keeping with single posturography testing conditions. Thus, Kapoula and Bucci [1] concluded that rather than suffering from a primary postural syndrome, dyslexic adolescents exhibit ocular fixation instability coupled with a particularly reduced capacity to maintain the angle of vergence at the required depth, resulting in the putative postural instability observed in this patient population. Unstable fixation may be due to attention fluctuation. Actively performing vergence eye movements engages their visual attention thus leading to improved postural stability. Rochelle et al. [2] also proposed that postural instability in dyslexics might be due to their diminished capacity to maintain attention.

Such interpretations are at variance with studies which suggest a postural deficiency in dyslexics. For example, Quercia et al. [3], and Pozzo et al. [4] suggested that there is a postural deficiency syndrome in dyslexia, that is an alteration of postural equilibrium accompanied by a deficit in the sensory processing of proprioceptive afferences from the legs and feet, and a deficit of visual information and extraocular proprioceptive input (see [3], [4]). Vieira et al. [5] used a double task (reading words of different colors) inspired from the well known Stroop test, introduced in 1935. They studied both dyslexics (age range of 8–16, mean age 12) before and after wearing prisms and proprioceptive soles for a period of 3 months as well as a control group of non dyslexics. The authors reported no difference between groups with respect to postural stability in the fixation control task. In contrast, for the double task, postural instability was greater in dyslexics than in controls; moreover, after the 3-month period of prismatic treatment combined with soles, this difference was resolved. Consequently, the Viera study [5] suggests that postural deficits in dyslexics are task specific and that prisms combined with soles are useful for improving postural stability. Note that posture recordings were performed without prisms and soles, both before and after the 3-month trial period. We will return to the use of prisms later. As far as the double task is concerned, another study from our group [6] combining posturography with the Stroop task was run on older adolescents. We used a series of words of different colors presented on a page. The participants had to keep themselves from reading the word and instead name the color of the ink in which the word was written – the task was similar but not identical to that used by Vieira et al [5]. The mean age of the adolescents studied by Kapoula et al. [6] was 15 years-old (14–17). The results demonstrated no deficit for the dyslexic versus the control group relative to basic postural parameters i.e. surface area of the center of pressure (CoP) excursions, the standard deviations of lateral and antero-posterior excursions, and its variance of speed. Subtle differences were found in the time frequency domain (wavelet analysis) suggesting relative inefficiency, yet without deterioration of stability. Thus, the existence of a postural deficit in dyslexics remains controversial.

We will now return to the prisms. One of the many hypotheses advanced in order to account for the origin of developmental dyslexia points toward a possible dysfunction of the cerebellum (e.g., see [7], [8]). To test this hypothesis, Brookes et al. [9] studied prism adaptation and showed that in most cases of dyslexia impairment of such adaptation does indeed occurs. The use of prisms as a tool for reducing some of the difficulties associated with reading has been studied by several research teams [10]–[14]. Motivated by the clinical use of prisms on postural behavior in dyslexia, the present laboratory study aims to assess the immediate effects of convergent prisms on posture in dyslexic and non-dyslexic teenagers. Prisms deviate the images and require a change in the ocular motor vergence angle. In healthy adults, use of convergent prisms is known to improve postural stability [15]. Whether a similar effect can be seen in adolescents (dyslexic or non-dyslexic) is not yet known. As mentioned in the study conducted by Vieira et al. [5], prisms were not used during posture recordings; prisms and proprioceptive soles were used as clinical treatment for 3 months. The present study aims to respond to the theoretical question of the immediate effects produced by convergent prisms and does not apply the prism configuration used in the Vieira study [5] even though the clinical configuration of the prisms used might also exhibit a convergent component. Other relevant laboratory studies on prisms and posture concern vertical prisms. Matheron et al. [16] and Matheron and Kapoula [17] used small vertical prisms in adults and reported that the effects on posture are complex, improving or deteriorating posture, depending on the eye wearing the prism (dominant *versus* non dominant). Indeed, while recording eye movements, Matheron et al. [18] showed that the quality of vergence eye movements that reduce more or less efficiently the disparity induced by the prism depends on the eye wearing the vertical prism: eye movements being more appropriate when the dominant eye wears the prism. Vertical prisms in children and their effect on posture were not examined in these above mentioned studies.

It should be noted that prisms modify the vergence angle thereby creating a conflict with the accommodation of the eyes. Accordingly, in the present study we therefore examined the effects of accommodation. To this end we used a negative spherical lense over each eye (−1 or −3 diopters). A change of accommodation produced by the lenses causes conflict with the vergence angle. The conflict technique used here aims to test the potential of each of these two initial catalysts (prism-induced vergence and lense-induced accommodation) in modifying postural stability in adolescents. One such methodology has been used in the past by our group (see [19]). It should be noted that because of reciprocal interactions between vergence and accommodation, the use of a prism will ultimately result in a slight modification in accommodation, and conversely a spherical lens accommodative change will also modify the vergence angle. These two interactions are described by the well known ratios of accommodative convergence to accommodation – AC/A – or convergent accommodation to convergence – CA/C – (e.g. [20], [21]). The results show that due to such conflicts, both prisms and spherical lenses, particularly those of small power, reduce postural stability and they do so similarly in both dyslexic and non-dyslexic adolescents. Wavelet analysis in the time frequency domain reveals increased spectral power for the prisms in both groups and suggests more difficulty with accommodation lenses in dyslexics as well as less efficient control of medium range frequencies hypothetically depending on the cerebellum. Taken together the results provide evidence for the importance of vergence and accommodation, such that when dyslexic and non-dyslexic teenagers are confronted by the conflict induced by prisms and lenses, a demonstrable deterioration in posture can be found.

## Materials and Methods

### Ethics Statement

The postural control investigation complied with the tenets of the Declaration of Helsinki and was approved by the local human experimentation committee, the “Comité de Protection des Personnes” (CPP) Ile de France VI (No: 07035), Necker Hospital in Paris, France. Written, informed consent was obtained from the participant's parents after the nature of the procedure had been explained.

### Subjects

Twenty-five emmetropic adolescents (5 females) were recruited from the College of Saint Sulpice in Paris, France. Fourteen were dyslexics (3 females, 11 males) in the age range of 13–17 years (15.6±0.9), and 11 non dyslexics (2 females, 9 males) in the age range of 13–16 years (15.1±1.0).

Dyslexics were admitted to this college precisely because they were known to be dyslexic. They underwent extensive examination, including neurological/psychological and phonological tests which were conducted during the course of the current year of the present study. For each participant, their rate of reading, text comprehension, as well as their capacity to read word/pseudo words was evaluated by using the L2MA battery [22]. This is the standard test developed by the applied psychology centre of Paris, and is used extensively in France. Inclusion criteria were: scores in these tests beyond two standard deviations; a normal mean intelligence quotient (IQ, evaluated with WISC III), i.e., between 85 and 115. Attention and concentration problems in the absence of hyperactivity were present in 6 of the dyslexics (3 with severe dyslexia and 3 with moderate); no teenager had dyspraxia. Such a population, including a few cases of teenagers with attention problems, is representative of the general dyslexic population. At secondary school, dyslexics followed the same educational program as the other pupils with the exception of additional classes for improving reading and orthographic skills. In parallel, they followed individual training with an orthophonist. However, reading difficulties persisted and were especially severe for 5 of the students. Problems with orthography were also present in 4 of the dyslexics.

Non-dyslexic adolescents had to satisfy the following criteria: no known neurological or psychiatric abnormalities, no history of reading difficulty, no visual stress or any difficulties with near vision. IQ and reading measurements could not be applied to these teenagers. It should be noted that there is no evidence that postural control depends on intelligence. Non dyslexics were selected by the director of their school on the basis of their overall school performance which includes their score in French (reading, understanding, and orthography), mathematics and foreign languages all of which were above the mean score of the class. It should also be noted that the reading score for non dyslexics was higher than that of dyslexics. Recruitment of non dyslexics adolescents based entirely on academic performance alone has been done previously by others [23]–[25].

### Platform characteristics

To measure postural stability, we used a force platform (principle of strain gauge) consisting of two dynamometric clogs (Standards by Association Française de Posturologie; produced by TechnoConcept, Céreste, France). Body sway was evaluated by computing the excursions of the center of pressure (CoP) measured over a period of 51.2 seconds; the equipment contained an Analog–Digital converter of 16 bits and the sampling frequency of the CoP was 40 Hz.

### Visual target

**Figure 1:**
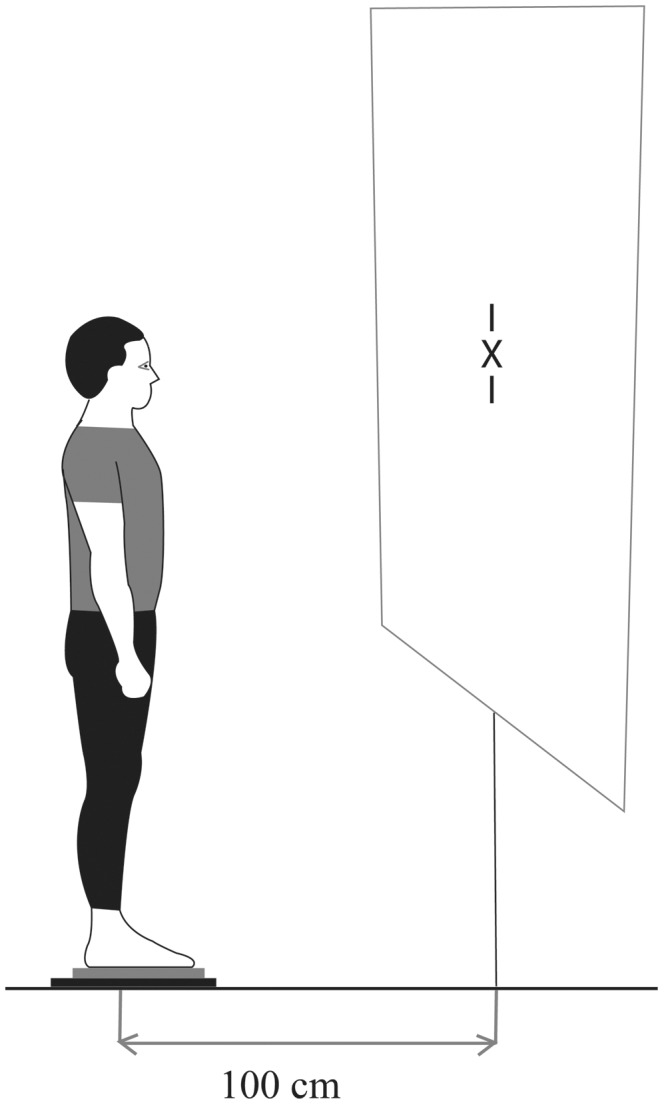
Illustrations of posturographic testing conditions. The subject viewed the “X” target on the screen at eye-level and from distance of 1 m.

The visual target was placed at eye level for each subject in upright stance on the force platform. A white wall was used to display the target along the vertical midline. The visual target was a picture with a little cross “x” (see Figure 1).

### Testing conditions

Subjects were placed on the force platform and were asked to fixate the “x” target in the straight ahead position for 51.2 seconds. The target was positioned at eye-level and at a distance of 100 cm (see Figure 1). There were four conditions: normal viewing (NV), accommodation with a negative spherical lens of −1 diopter in front of each eye (ACCOM1), accommodation with a negative spherical lens of −3 diopters in front of each eye (ACCOM3), vergence with a convergent prism of 8 diopters in front of each eye (PRISM).

For the condition ACCOM3, the use of a −3 diopter spherical lens in front of each eye was aimed at bringing the accommodation to 33 cm, conflicting with the accommodation required by the physical distance of the target on the screen (1 m i.e., −1 spherical diopter). Such spherical negative lenses caused blur and stimulated accommodation, and subsequently accomodative convergence. The normal AC/A ratio [Accommodative/Convergence (in prism Diopters)/Accommodation (in spherical diopters)] is 3–4 (see [26]). Mutti et al. [27] showed that the AC/A ratio does not change significantly with age between 6 to 14 years. Therefore, given the −3 diopters lenses we used, the expected accommodation would be −4 spherical diopters, and the expected accommodative convergence would be 10–12 prisms diopters, which approximately corresponds to 5–6° of convergence (1 prism diopter = 0.57°). Note that the vergence angle while fixating the target naturally at a distance of 1_m should be about 3.4° and at a distance of 33 cm around 9°. Thus, the modified vergence angle will also be in conflict with the vergence angle required by the physical distance (3.4° for 1_m viewing distance).

For the condition ACCOM1, the required accommodation for vergence at 1_m is −1 spherical diopter. By inserting a −1 spherical diopter lens in front of each eye we induce an accommodative requirement of 2 diopters. Thus, the accommodation requirement and the potential conflict with the vergence angle were less than in the ACCOM3 condition; considering the accommodation vergence interaction and the hypothetical AC/A ratio of 3–4, one would expect the vergence angle to also be modified to 6–8 prism diopters (corresponding to 3–4°), which is close but not identical to the vergence angle required by the physical distance of 1 m. This conflict is also smaller for the ACCOM1 than for the ACCOM3 condition.

The prism condition was performed as follows: the target on the screen was always positioned at a distance of 1 m. An 8 diopter convergent prism was placed in front of each eye. Such a prism should stimulate convergence of the eyes by 16 prism diopters approximately corresponding to the vergence required by a target presented at a distance of 33 cm (9°). The initial conflict here was between the vergence angle induced by the prism and the vergence angle required by the physical distance; also a conflict between the convergence angle induced by the prism and the accommodation corresponding to the physical distance of 1_m. Again, because of the reciprocal interaction between convergence and accommodation, the prism induced convergence could modify the accommodation. The normal CA/C ratio being 0.1, i.e., 10 prismatic diopters of convergence would cause approximatively 1 spherical diopter of accommodation; one would expect that prisms bring accommodation to −2.6 diopters, i.e., a level still different from that required by the 1_m physical distance. Thus, for all conditions except the NV, there was a mismatch between both vergence and accommodation as they are related to each other, as well as between vergence and accommodation and conflicts relative to values required by the physical distance.

Throughout the experiment the participants wore an ophthalmologic spectacle frame upon which were inserted the spherical lenses or the prisms. The Fresnel prisms used did not alter visibility of the fixation target. The order of the conditions was counterbalanced. Between conditions, the participants sat on a chair and one minute rest period was provided. We asked the participants to close their eyes before standing up. Then, upon a “go” signal given by the investigator, the children opened their eyes and the posturographic recordings were initiated. No preliminary training was necessary, as all teenagers readily performed the sequence of conditions.

### Basic postural parameters

We analyzed the surface of the CoP excursions, the standard deviations of lateral (SDx) and anteroposterior (SDy) body sways and the variance of speed. The surface area was measured with the confidence ellipse including 90% of the CoP positions sampled eliminating the extreme points [28].

### Frequency analysis

We also applied a wavelet non linear analysis to study frequency in the time domain. Applied to CoP displacements, the wavelet transform elaborates a time-frequency chart of body sway [29], [30]. The wavelet analysis used by the software is a continuous one. The mother wavelet used is Morlet. The time-frequency plane's principle advantage is its double resolution (time and frequency). Thus, the fact that the spectrum of the body sway is not constant over time is shown. The wavelet analysis was applied on the anteroposterior and mediolateral sway data. The spectral power was calculated for the frequency bands 0.05–0.5 Hz (F1), 0.5–1.5 Hz (F2), higher than 1.5 Hz (F3) on the anteroposterior and mediolateral sways as power indices (PIy and PIx, respectively). The hypothetical physiological significance of the spectral power of different bands is as follows: 0–0.5 Hz visual-vestibular [31], [32], [33], 0.5–1.5 Hz cerebellar [33], >1.5 Hz reflexive loops [30], [34]. As a rule, power in the higher band (F3) is minimal in healthy subjects during quiet standing, but it can be observed with aging, and postural pathology, or in dynamic postural conditions [30]. Moreover, the canceling time (CT) of each frequency band was also calculated for the antero-posterior (CTy) and mediolateral (CTx) sway, i.e., the total time during which the spectral power of the body sway for the frequency range was cancelled by the posture control mechanisms; the longer the canceling time of a frequency band, the better the posture control [29], [30]. The fact that a certain frequency has its power reduced to zero over a period of time shows that there has been a successful action of the postural control system since the overall entropy of the sway is reduced. While most healthy subjects exhibit these zero power instances in their postural sway spectrum, the pathological subjects cannot. How the cancelled frequencies are “chosen” by the postural control system is not known, but it may be assumed that the choice criterion is the minimization of muscular effort required for controlling the sway. The postural instability index (PII) which quantifies the postural performance by taking into account the two precedent indices (PI and CT), was also calculated [29], [30] and yielded the following results: PII = SxSyPI(F1, F2, F3)/CT(F1, F2, F3). In healthy adults and during the single quiet stance task, the PII is close to unity (see [30]). This additional analysis and associated parameters were obtained with the software PosturoPro (Framiral, Cannes, France, www.framiral.fr).

### Statistical analysis

After the log transformation of the data (due to differences in variance), a mixed ANOVA design was used, with a main factor, the different viewing conditions with 4 levels (NV, ACCOM1, ACCOM3 and PRISM), and one inter-subject factor with 2 levels, subjects with dyslexia or not. The post hoc comparisons were done by Fischer's PLSD test. P<0.05 was considered significant.

## Results

### Results for basic posture parameters

Means and standard errors are shown in Table 1 for each group of subjects (Dyslexics and Controls) and for each condition (NV, ACCOM1, ACCOM3 and PRISM) for all basic postural parameters, i.e. the surface area of CoP excursions, the standard deviation of lateral and anterior-posterior body sway, and the variance of speed.

**Table 1:** Postural stability measurements in quiet stance (51.2 seconds duration) for basic parameters.

	Conditions			
	Normal Viewing	Accommodation 1	Accommodation 3	Convergent Prisms 8
**Surface of CoP (mm^2^)**				
Dyslexics	164.35±26.06	272.84±35.50	251.95±40.72	282.27±39.66
Controls	223.91±39.23	295.61±79.92	216.77±27.76	337.49±52.46
**Standard deviation of lateral sway (mm)**				
Dyslexics	2.84±0.33	3.80±0.38	3.54±0.43	3.29±0.31
Controls	3.81±0.49	3.85±0.55	3.14±0.30	3.95±0.44
**Standard deviation of A/P sway (mm)**				
Dyslexics	4.28±0.34	5.68±0.83	5.28±0.35	6.15±0.58
Controls	4.35±0.24	4.96±0.79	4.99±0.37	6.51±0.62
**Speed variance (mm^2^/s^2^)**				
Dyslexics	44.93±8.49	58.58±10.94	46.78±8.11	63.94±8.93
Controls	62.33±12.43	66.85±10.11	67.28±9.02	76.05±11.27

Means and standard errors of the surface of CoP, standard deviations of lateral and of anteroposterior body sway, and variance of speed for each conditions i.e., normal viewing (NV), accommodation with a negative spherical lens of −1 diopter in front of each eye (ACCOM1), accommodation with a negative spherical lens of −3 three diopters in front of each eye (ACCOM3), and with convergent prism of 8 diopters in front of each eye (PRISM) for 14 dyslexics and 11 non dyslexic adolescents.

There was no group effect or interaction between the inter-subject factor and the viewing conditions for all parameters. In contrast, there was a main effect of the viewing conditions on the surface area of CoP (*F*(3,69) = 3.72, p = .015), and on the standard deviation of anterior-posterior body sway (*F*(3,69) = 5.85, p = .001), but not on the standard deviation of lateral body sway (*F*(3,69) = 1.05, p = .376), nor on the variance of speed (*F*(3,69) = 2.60, p = .060). Next will be presented the post-hoc analysis for the accommodation and prisms conditions.

### Effects of accommodation

**Figure 2:**
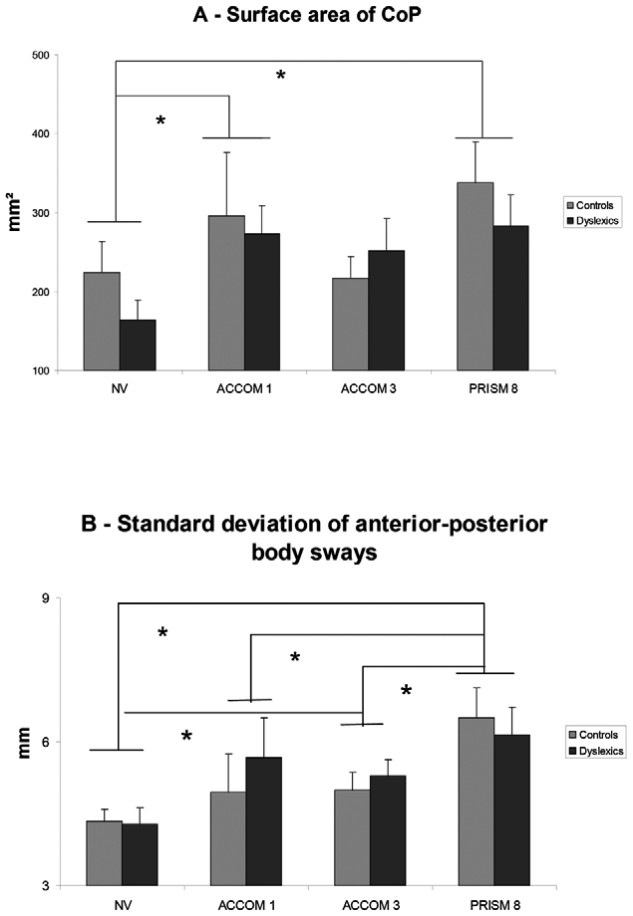
Effects of spherical lenses and prisms on postural parameters. Means of the surface area of CoP (**A**) and of the standard deviations of anteroposterior (SDy) body sway (**B**) for both, Dyslexic and Control adolescents. Error bars represent the standard error. Asterisks indicate significant differences (p<0.05).

The Fischer's PLSD post hoc showed significant increase of the surface area of the CoP for the ACCOM1 compared with the NV (p = .046). Interestingly, lenses of −3 diopters in the ACCOM3 condition did not induce significant increase of surface of the body sway (see Figure 2-A).

In contrast, the ACCOM3 condition caused a significant increase of the standard deviation of anterior-posterior body sway compared to NV (p = .048), see Figure 2-B.

### Effects of prisms

The PRISM condition increased the surface significantly compared to the NV condition (p = .001); moreover it also increased the standard deviation of anterior-posterior body sway relative to NV (p = .0001). See Figure 2-A.

### Comparison Accommodation vs. Prism conditions

The standard deviation of anterior-posterior body sways for the PRISM was higher than that for either of the accommodation conditions (respectively p = .008 and p = .037, see Figure 2-B).

To summarize, both, the lenses and the prisms modified spatial but not temporal parameters of postural control and they did so similarly for dyslexic and non dyslexic teenagers.

### Results for frequency analysis

This section presents the results of the wavelet analysis applied on the CoP displacements (see Methods). Table 2 shows the group means and the standard errors of the postural instability index (PII), the canceling time of each frequency band and the spectral power for different frequency bands for anteroposterior and medio-lateral sways. Results are shown for each of the four conditions and for each group of subjects.

**Table 2:** Values of postural parameters from wavelet analysis.

		Controls	Dyslexics
**Wavelets PII**	*NV*	1.85±0.19	1.40±0.17
	*ACCOM1*	1.63±0.18	1.46±0.11
	*ACCOM3*	1.62±0.20	1.67±0.12
	*PRISM*	1.80±0.15	1.82±0.10
PIy (mm^2^*10^6^) 0–0.5 Hz	*NV*	73.58±1.80	70.91±1.04
	*ACCOM1*	73.46±1.93	71.17±1.28
	*ACCOM3*	70.70±1.69	74.82±1.41
	*PRISM*	74.26±2.15	76.33±1.24
**0.5–1.5 Hz**	*NV*	61.40±2.15	58.39±1.70
	*ACCOM1*	61.26±2.03	59.25±1.10
	*ACCOM3*	59.60±1.60	60.77±1.46
	*PRISM*	62.50±1.61	63.27±1.17
**>1.5 Hz**	*NV*	45.46±2.08	41.73±1.63
	*ACCOM1*	44.36±1.71	42.73±1.20
	*ACCOM3*	42.99±1.88	44.2±1.36
	*PRISM*	46.51±1.56	46.70±1.11
PIx (mm^2^*10^6^) 0–0.5 Hz	*NV*	68.80±2.88	63.61±2.55
	*ACCOM1*	67.05±2.98	64.10±1.84
	*ACCOM3*	67.09±2.35	67.67±2.02
	*PRISM*	67.16±2.20	68.12±1.15
**0.5–1.5 Hz**	*NV*	58.56±2.48	52.77±2.41
	*ACCOM1*	56.40±2.80	53.32±1.88
	*ACCOM3*	56.56±2.19	55.29±1.59
	*PRISM*	55.30±1.98	56.39±1.04
**>1.5 Hz**	*NV*	41.21±2.81	35.22±2.33
	*ACCOM1*	38.73±2.76	35.72±1.87
	*ACCOM3*	39.60±2.67	39.00±1.88
	*PRISM*	39.00±1.88	39.75±1.10
**CTy (s) 0–0.5 Hz**	*NV*	0.41±0.07	0.56±0.09
	*ACCOM1*	0.64±0.19	0.57±0.08
	*ACCOM3*	0.71±0.15	0.51±0.12
	*PRISM*	0.50±0.09	0.45±0.09
**0.5–1.5 Hz**	*NV*	1.33±0.20	0.92±0.12
	*ACCOM1*	1.48±0.22	0.94±0.09
	*ACCOM3*	1.66±0.28	0.89±0.14
	*PRISM*	1.59±0.23	1.11±0.14
**>1.5 Hz**	*NV*	0.01±0.00	0.01±0.01
	*ACCOM1*	0.01±0.00	0.01±0.01
	*ACCOM3*	0.01±0.01	0.00±0.00
	*PRISM*	0.01±0.00	0.01±0.01
**CTx (s) 0–0.5 Hz**	*NV*	0.92±0.29	1.33±0.21
	*ACCOM1*	1.46±0.76	0.99±0.15
	*ACCOM3*	1.04±0.33	0.94±0.17
	*PRISM*	0.73±0.16	0.86±0.19
**0.5–1.5 Hz**	*NV*	0.97±0.12	0.53±0.12
	*ACCOM1*	0.84±0.18	0.66±0.18
	*ACCOM3*	1.22±0.28	0.50±0.10
	*PRISM*	1.19±0.21	0.78±0.12
**>1.5 Hz**	*NV*	0.01±0.00	0.01±0.01
	*ACCOM1*	0.01±0.01	0.00±0.00
	*ACCOM3*	0.02±0.01	0.00±0.00
	*PRISM*	0.01±0.00	0.01±0.01

Means and standard errors of PII, and PI and CI for each plane (PIy, PIx, CIy and CIx, respectively) for each frequency band (0.05–0.50 Hz, 0.50–1.50 Hz, 1.50–10.00 Hz) and for each condition, i.e. normal viewing (NV), accommodation with a negative spherical lens of −1 diopter in front of each eye (ACCOM1), accommodation with a negative spherical lens of −3 three diopters in front of each eye (ACCOM3), and with convergent prisms of 8 diopters in front of each eye (PRISM); data from 14 dyslexics and 11 non dyslexic adolescents.

### Effect of prisms

**Figure 3:**
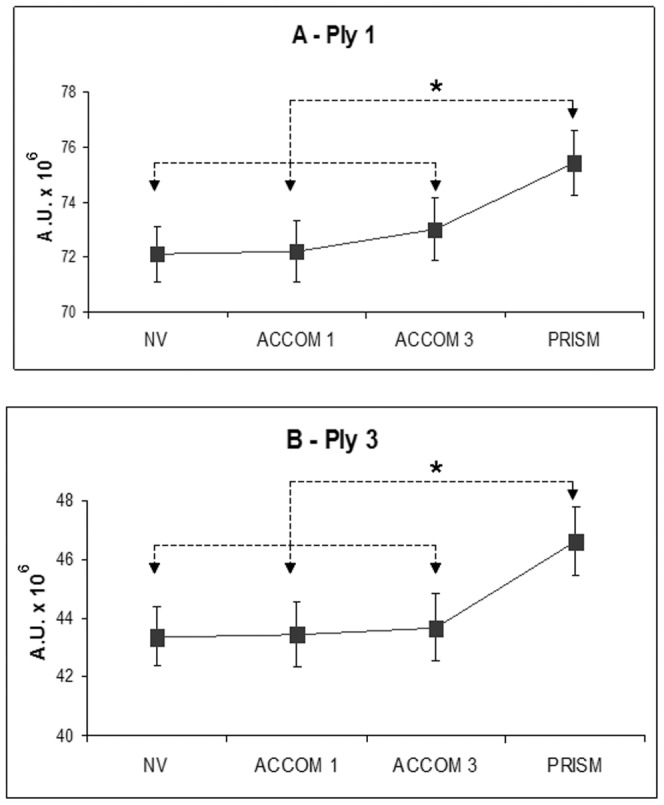
Effects of prisms. The power index of the anteroposterior body sway for the low frequency band (A) and for the high frequency band (B) is higher for the PRISM condition relative to all other conditions. Bars represent the standard error, asterisks indicate significant difference (p<0.05).

There was a main effect of the condition on the power index for the low frequency band (PIy 1, *F*(3,69) = 3.03, p = .035) and for the high frequency band (PIy 3, *F*(3,69) = 2.87, p = .043) on the antero-posterior sway. The Fisher's PLSD post hoc showed significant increase of PIy 1 and of PIy 3 for the PRISM condition compared to the NV condition (p = . 011 and p = .019, respectively), also compared to the ACCOM1 condition (p = .013 and p = .033, respectively) and to the ACCOM3 condition (p = .017 and p = .019, respectively (see Figure 3A,B).

### Effect of group

**Figure 4:**
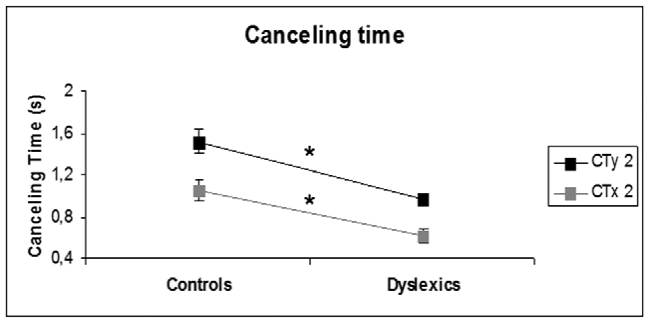
Effects of group in dyslexics vs. controls on the canceling time. Cancelling time of the medium frequency band (0.5–1.5 Hz) for the anteroposterior (CTy 2) and the mediolateral (CTx 2) body sway is shorter for dyslexics. Bars represent the standard error, asterisks indicate significant difference (p<0.05).

There was a main effect of group for two of the parameters elaborated from the wavelet analysis: the canceling time of medium frequency band (0.5–1.5 Hz, hypothetically controlled by the cerebellum) was shorter for dyslexics than non dyslexics; this was the case for both the antero-posterior (CTy) and the mediolateral (CTx) sway (respectively, F(1,23) = 7.16, p = .014 and F(1,23) = 5.94, p = .023, see Figure 4).

### Group condition interaction

**Figure 5:**
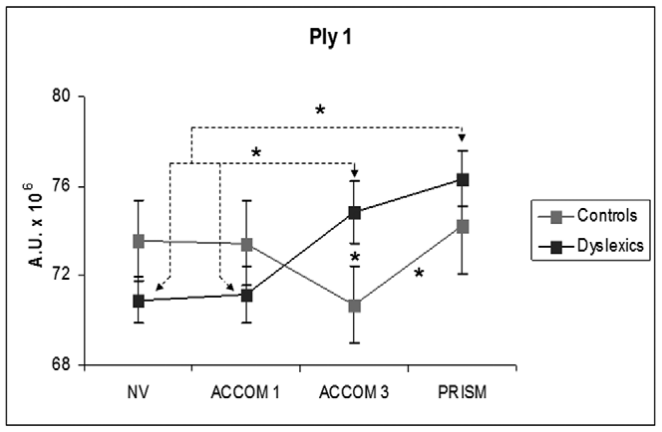
Interaction between groups and tasks for the PIy 1. Power index of anteroposterior body sway for the low frequency band (0–0.5 Hz) believed to be control by vision. Bars represent the standard error, asterisks indicate significant difference (p<0.05). Spherical lenses of −3 diopters used in ACCOM3 condition to induce strong accommodation increases considerably PIy 1 for dyslexics but decreases for non dyslexics. On the other hand, non dyslexics show similar PIy for all other viewing conditions while dyslexics show increase PIy for frequency also for the PRISM condition.

A significant interaction between group and condition was found on the parameter PIy 1, i.e., the power index for the low frequency band on the antero-posterior sway (PIy 1, *F*(3,69) = 4.02, p = .011 (see Figure 5). The Fisher's PLSD post hoc showed significant increment of the PIy 1 in dyslexics *versus* controls for the ACCOM3 condition (p<0.05). Comparisons within each group show the following. In dyslexics, the PIy 1 is higher in ACCOM3 and in the PRISM conditions compared to the NV (p = . 014 and *p* = .0009, respectively), and also compared to the ACCOM1 condition (p = .022 and p = .001, respectively). In contrast, for the controls, this parameter is lower in the ACCOM3 condition relative to the PRISM condition (p = .046).

To summarize, the main effects revealed by wavelet analysis are for antero-posterior sway: the PRISM condition shows the highest spectral power for both groups, in the ACCOM3 condition the power index is higher in dyslexics than non dyslexics; canceling time of the medium range frequency of both, antero-posterior or mediolateral sway are shorter for dyslexics than for non dyslexics. The statistically significant effects from both sections are summarized in Table 3.

**Table 3:** P-values from ANOVA.

	*p* Group	*p* Task	*p* Task*Group
**Surface mm^2^**	0.675	0.015*	0.366
**Sdy (mm)**	0.830	0.001*	0.643
**Sdx (mm)**	0.411	0.376	0.213
**Speed Variance (mm^2^/s)**	0.146	0.059°	0.699
**Wavelets PII**	0.370	0.194	0.177
**Ply 1**	0.855	0.035*	0.011*
**Ply 2**	0.646	0.069°	0.269
**Ply 3**	0.538	0.043*	0.224
**Plx1**	0.489	0.618	0.261
**Plx2**	0.277	0.921	0.239
**Plx3**	0.313	0.591	0.264
**CTy1**	0.673	0.406	0.385
**CTy2**	0.014*	0.246	0.432
**CTy3**	0.471	0.834	0.685
**CTx1**	0.987	0.237	0.240
**CTx2**	0.023*	0.197	0.227
**CTx3**	0.166	0.948	0.105

On the studied postural parameters for group (dyslexics vs. controls), task (normal viewing task, accommodation with a negative spherical lens of −1 and −3 diopters, vergence with a convergent prism of 8 diopters) and group-task interaction effects. Asterisk indicates significant difference (p<0.05) and circle indicates marginally significant effects.

## Discussion

The study provides several new results:

1. Spherical lenses modifying accommodation decrease postural stability (increase of standard deviation of anteroposterior body sway and/or of the surface); paradoxically lenses of weaker power are more disturbing.
2. Convergent prisms also destabilize posture, increasing the surface of body sway and of standard deviation of anteroposterior body sway.
3. All effects on basic parameters of posture are similar for the two groups of teenagers and there is no significant difference between dyslexics and non dyslexics.
4. In the frequency domain, the spectral power for the antero-posterior body sway (low and high frequency bands), is higher for the PRISM condition relative to all other conditions, again with no difference between dyslexics and non dyslexics.
5. Modification of accommodation with the three diopter spherical lenses decreases the power index of antero-posterior sway for non dyslexics but increases it for dyslexics to a level as high as that for the prism condition.
6. The canceling time of medium frequency range, believed to be controlled by the cerebellum, is shorter in dyslexics, indicating shorter periods of optimal control.

Each of these results will be discussed below.

### Spherical lenses - accommodation and posture instability

As mentioned in the introduction, the spherical lenses used cause blur stimulating accommodation changes by 2 or 4 spherical diopters in the ACCOM1 and ACCOM3 conditions respectively. Such change of accommodation creates a conflict with the vergence angle corresponding to the physical distance (1_m). Due to reciprocal interaction, accommodation changes will induce some changes in the vergence angle, but some conflict will always remain. Although neither the accommodation nor the vergence angle were objectively measured in the study, it is certain that such changes occurred. Here we objectivised the consequences of such changes on postural control. The results showed that changes of the accommodation and to a less degree of vergence have a direct effect on the basic parameters of posture of teenagers. To our knowledge, prior studies of the impact of accommodation on posture are scarse. Early in the 1980's, some studies reported effects of blurring of the images (corresponding to accommodation cues) on postural control (see [35], [36]). Our observations in teenagers expand this conclusion.

By what mechanism does the accommodation driver act on postural control? The most relevant study is that of Han and Lennerstrand [37]. These authors reported that vibration of cervical muscles changed the dynamics of accommodative vergence. Therefore, we propose that accommodation, and accommodative vergence induced by the lenses could modify activity in the neck muscles that are known to be a major relay for postural control. Visual, oculomotor and somatosensory inputs are all integrated in the superior colliculus, the cerebellum and the vestibular nuclei (see [38]), and influence the vestibuloocular, vestibulospinal and reticulospinal systems that are involved in postural control (see [39]). At the perceptual level, accommodation provides a cue related to depth. Thus, it is interesting that the major effect on posture in the ACCOM3 condition is seen on the standard deviation of anteroposterior body sway. This effect is plausible physiologically and occurs for the strong power lenses (−3 spherical diopters). Next, we will discuss the paradoxical observation of increase of the surface of body sway for the weaker spherical lenses relative to the control condition.

We propose that posture instability (increase of surface) results when the accommodative response to spherical lenses is inappropriate, e.g., too high or too weak relative to the power of the lens. A similar mechanism has been proposed by Matheron et al. [16], [18] in their adult studies dealing with small vertical prisms. We suggest that with spherical lenses of −3 diopters, teenagers can mobilize robust compensatory accommodative responses. Indeed their accommodative abilities are robust [26]. High gain accommodative ability is perhaps reinforced by dense, every day activities such as watching movies, play stations (on PCs, I-Pod, I-Phone). The condition ACCOM1, using 1 diopter lenses, requires a subtle, moderate, modification of the accommodation. Most likely teenagers fail to perform such adjustments; perhaps they over accommodate and this increased the conflict thereby leading to more posture instability. As mentioned in the Introduction, the studies of Mutti et al. [27] show that the AC/A ratio does not change significantly with the age of children from 6 to 14 years old. Yet, the AC/A ratio can increase if the interpupillary distance of the subjects increases (see [40]). To our knowledge, objective measures of the accommodation and of vergence are missing in the literature as well as in the present study. The frequency analysis results to be discussed later indicate significant differences between dyslexics and non dyslexics for the ACCOM3 condition even though the two groups did not differ in terms of basic parameters; presumably the same stability can be obtained via different mechanisms, and accommodation could be more highly demanding for dyslexics.

Moreover, if, as we suggest, accommodative vergence induced by the lenses could modify activity in the neck muscles (see [37]) that are known to be a major relay for postural control, a kinematic analysis using a motion tracking system, instead of simple posturography recordings, would be of interest. Kinematic analysis has been used in children and adolescents, providing accurate descriptions of inter-segmental orientation in multiple postural tasks, an important analysis not possible with posturography platforms (see [41]).

### Effects of prisms

The convergent prisms used called the eyes to converge significantly, as if they were fixating at 33_cm, creating a conflict with the vergence required by the physical distance which was 1_m. Convergence of the eyes (even though not measured) occurred. A conflict also exists between the vergence angle and accommodation. Here again, because of the reciprocal interaction between convergence and accommodation, convergence induced by the prisms would modify the accommodation by an amount determined by the AC/A ratio of the teenagers (see Methods). A prior study in healthy adults [15] showed improvement of posture stability with convergent prisms of 5 diopters per eye, despite conflicts. Note that the study in adults compared 200_cm versus 40_cm distances, while in the present study we used 100_cm versus 33_cm for which conflict is perhaps more intense. Kapoula and Lê [15] attributed the improvement of the posture in adults to the benefit from efferent and afferent oculomotor signals related to convergence of the eyes, improving the stability despite conflicts. The observations for teenagers show a different opposite pattern indeed, as the surface of body sway increased as well as the anteroposterior body sway. Perhaps teenagers do not produce well matched vergence accommodation responses when wearing the prisms. Another complementary possibility is that teenagers do not yet rely as much as adults on internal ocular motor signals and thus cannot yet benefit from the convergence to stabilize posture. In other words, the conflict between vergence and accommodation prevails relative to the benefit from strong convergence described in adults by Kapoula and Lê [15]. Furthermore, vision has an important effect in adolescents' body stabilization, and adolescents do not use proprioceptive inputs optimally; Viel et al. [42] suggested that the mechanisms underlying postural control are still maturing during adolescence, which might constitute a transient period of proprioceptive neglect in sensory integration of postural control. Further studies with objective measures of accommodation and of vergence combined with posturography would be of interest. In conclusion, the study shows that convergent prisms have destabilizing immediate effects on posture of teenagers. The frequency analysis in the time domain showed increased spectral power for both low and high frequency ranges in the PRISM condition relative to all other conditions. This additional analysis clearly indicates that prisms are highly demanding, requiring increased energy to maintain postural stability for all teenagers.

### No differences in basic posture parameters between dyslexic and non dyslexic teenagers

The comparison between groups for all conditions shows irrevocably no differences between dyslexic and non dyslexic teenagers on the basic parameters of posture control. The evidence provided here is in line with our prior studies [1], [6] and argues for no systematic deficits on posture in dyslexics teenagers. As discussed by Kapoula et al. [6] a recruiting bias (school versus clinic) might explain controversies in the literature, as well as differences in age of subjects studied [42] and differences of categories/comorbidity with dyslexia (see [2], [43]–[47]). Other authors have proposed that evaluating postural control in challenging postures, instead of during a simple “standing upright” condition, may be necessary to detect alterations of postural control (e.g. [34], [48]). This point was beyond our aim, i.e. to test first the effects of convergent prisms and spherical lenses during quiet upright stance, nevertheless reminds of interest. Other controversies are based mostly on the study of basic parameters of posture. As other studies point to the need to examine further parameters including frequency analysis and diffusion analysis (see [6], [49], [50], we also studied frequency aspects with wavelets analysis. The subtle differences that were found will be discussed later.

### Prisms induce higher spectral power

The major effect of the present study is that the PRISM condition increases the power indices for the low and high frequency ranges for the antero-posterior body-sway (see Figure 3A and 3B). This global effect which concerns both groups provides further evidence, in keeping with the basic posture parameters, that prisms for teenagers are highly demanding. They deteriorate postural stability (increase of surface) and amplify the spectral power for both the low and high frequency ranges that are believed to be controlled by vision, and rapid sensorimotor loops, respectively.

### Strong accommodation increases power frequency for dyslexics

For the −3 diopters spherical lens condition, the basic posture parameters showed increase of antero-posterior body sway relative to other conditions similarly for both groups. Yet, the wavelet analysis indicates that such an increase is subtended by different physiologic mechanisms for the two groups of teenagers: for non dyslexics the power index decreases (e.g., relative to normal viewing condition) while for dyslexics it increases. Thus, dyslexics require greater energy expenditure in order to achieve similar postural behaviors in terms of antero-posterior sway. These results indicate that dyslexics might have more difficulty with strong accommodative requirements than non dyslexics. This is congruent with recent studies of children with reading difficulties who were found to have reduced accommodative amplitude assessed by optometric tests (see [51], [52]).

### Time of optimal posture control is shorter for dyslexics

The wavelet analysis revealed shorter cancellation times for dyslexics with respect to the medium frequency band which is hypothesized to be controlled by the cerebellum; cancellation time was shorter for both lateral and antero-posterior body sways. This observation is compatible with a more general theoretical framework suggesting a cerebellar deficit in dyslexics as being responsible for various difficulties encountered in this patient population with reading, writing and spelling (see [8], [53], [54]). The above observation is also compatible with our prior study comparing postural control in dyslexic and non dyslexic teenagers while doing the Stroop test [6]. Despite their increased difficulty with such a test, basic parameters of posture stability as measured in dyslexics and non dyslexics were entirely comparable with the exception of the cancellation time in the medium frequency band which were again shorter for dyslexics. In response to the mounting evidence provided by these data, we adduce the hypothesis of the mild inefficiency of cerebellar control of posture in dyslexia. However, such a hypothesis emphasizes the efficiency aspect, dyslexics successfully perform the same postural behaviors as non dyslexics but with greater energetic expenditure. It might also be observed that in more demanding conditions such as balance or dynamic conditions dyslexics differ more clearly from non dyslexics (see [53]). The quiet stance test used here is one of the most common postures humans use to interact with their environment; this test implies multisensory integration and successful stabilization of the body's many components. Yet, this test alone is not sufficient to diagnose postural pathologies. In terms of research however, the quiet stance test has been used to evaluate the effects of prolonged treatment with prisms and soles in dyslexia (e.g. [55]).

### Clinical implications

Our study highlights some of the negative effects that convergent prisms can have on postural control in dyslexic and non dyslexic teenagers. Prisms and spherical lenses deteriorate stability and strong accommodation requirements require more energy for dyslexics whose postural control is less efficient than for non dyslexics. Clinical practice often times involves precisely the use of such prisms in conjunction with some convergent action combined with proprioceptive soles [5]. As mentioned above, prisms change not only ocular vergence but also the link with accommodation. Although we cannot exclude *de facto* the benefits which might accrue to some adolescents with respect to the use of convergent prisms, especially adolescents exhibiting some kind of postural syndrome, the present study emphasizes the possible negative effects of prisms on posture control as previously reported with a lower prism diopter (e.g. [16], [56]–[58]). Objective and regular measures of vergence, accommodation and posture, all of which accompany such clinical treatment modalities, could help to improve our understanding of the causal mechanisms of action at work in postural control and eventually strengthen the utility of such treatments. A final point to be made includes the distinction between immediate and long-term effects. The present study has dealt with immediate effects as projected over an interval of 51.2 seconds. How does prolonged exposure to vergence accommodation conflicts ultimately become helpful? This question among others will require further study and analysis. Do the initial negative effects of prisms possibly revert to positive effects improving posture and at what precise period during the course of treatment? Studies of the respective role of prisms, soles and of their interaction are also important.

Beyond the issue of immediate versus long-term exposure effects there are still important questions concerning the existence of postural pathologies in dyslexia and the necessity for prism treatment. Rochelle et al. [2] note that apparent deficits in postural control in developmental dyslexia can be mediated by inclusion of children with hyperactivity. Brookes et al. [59] suggest that mixed results on balance difficulties may be attributable to variability in balance tasks, balance measurement, participant age, and inclusion of comorbid disorders such as ADHD.

The quiet stance posture test used here is not a diagnostic or therapeutic tool but rather a research tool; a similar test has been used to evaluate the effects of prism-sole treatments on dyslexia [55]. Given the negative nature of the immediate effects of prisms and lenses on the posture control of dyslexic and non dyslexic teenagers, and given the difficulty for accommodation experienced by dyslexics, we are inclined to suggest caution and regular follow ups when using prisms as a potentially viable treatment modality in dyslexic teenagers.
